# Supplementary material for: Rad6 Regulates Conidiation by Affecting the Biotin Metabolism in Beauveria bassiana
Source: J Fungi (Basel). 2024 Aug 28;10(9):613. doi: 10.3390/jof10090613 (PMC11433481; doi:10.3390/jof10090613)
Supplement: Supplementary file 1 [file jof-10-00613-s001.zip › Supplemental file S1.pdf]

Supplementary Material for *Journal of Fungi*

Rad6 Regulates Conidiation by Affecting the Biotin Metabolism in *Beauveria bassiana*

Yuhan Guo, Haomin He, Yi Guan* and Longbin Zhang*

Fujian Key Laboratory of Marine Enzyme Engineering, Fuzhou University, Fuzhou, Fujian, 350108, China

Correspondence: gy@fzu.edu.cn and longbin_z@163.com

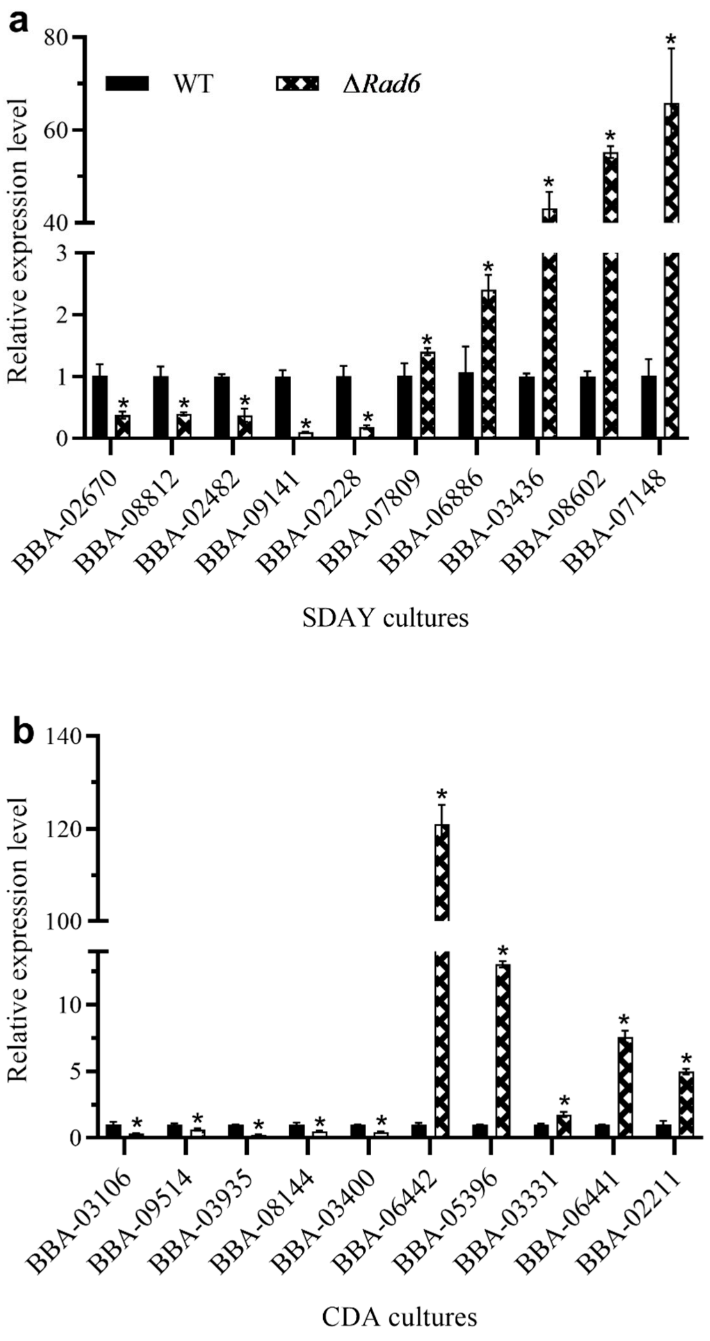

Figure S1. RT verification of SDAY (a) and CPZ (b) boards.The primers utilized for dual identification are detailed in Table S2 below.

Table S1. Paired primers designed for constructing deletion mutants and complementary mutants of *rad6* in *B. bassiana*.

Primers	Paired sequences (5′–3′)*	Purpose
*Rad6*up-F/R	TGGGCCCGGCGCGCCGAATTCCATTGCGGTAAGACTGGAT<br>/ TGG CTGCAGGTCGACGGATCCAAAGAGGTTATGCGTTGTTG	Cloning *rad6* 5′-end (1541 bp) for homogenous recombination
*Rad6*dn-F/R	GACCCATGGCTCGAGTCTAGATCCGATACCCGATAATGT<br>/ GGTGGTGG TGGCTAG GGTTAACCAAACGTACCTCCTCCCT	Cloning *rad6* 3′-end (1553 bp) for homogenous recombination
*Rad6*cp-F/R	GGGGACAAGTTTGTACAAAAAAGCAGGCTTGCGACCTGATCCTTTGT<br>/ GGGGACCACTTTGTACAAGAAAGCTGGGTCCTTGCGTGAGACCTTCC	Cloning full-length *rad6* (2712 bp) for complementation

* Underlined sections represent the segments used in homologous recombination for both *rad6* 5' and 3' fragments (used for targeted gene disruption) or for gateway exchange (targeted gene complementation). Double-underlined sections indicate the specific restriction enzyme sites (*Eco*RI/*Bam*HI and *Xba*I/*Spe*I) employed in the homologous recombination process.

Table S2. Paired primers used for assessing the reliability of transcriptome data in *B. bassiana* through qPCR analysis.

Gene	Tag loci*	Annotation	Paired primer sequences (5′–3′)
**SDAY transcriptome**			
actB	BBA_04860	β-actin	GGCAACATTGTCATGTCTGG/TTTGCTGGAAGGTGGATAGG
	BBA_02670	hypothetical protein	TTCGAGCAGTTGATGGACAC/ACGGTTTGACGGTATCTTGC
	BBA_08812	secreted lipase 1 precursor	ACGTCGATGGCACCTTATTC/CGAGGGTCTGGAAGCTAGTG
	BBA_02482	carboxypeptidase Y	TTCTCTGTGTCTGCCCTCCT/TAGTCCGCCAGGTAACCATC
	BBA_09141	hypothetical protein	CGCACTCGACAAGGTGACTA/GACGCTCCACGGAATAGAAG
	BBA_02228	hypothetical protein	CCCAGCAGTCGTAATGTCCT/AGGGACCAAGAGGATGGAGT
	BBA_07809	protease S8 tripeptidyl peptidase I (cln2)	CCCAATTTCGGAAAGCATTA/GCTTCTGGGGTGGTGATAGA
	BBA_06886	acyl transferase domain-containing protein	TCGTCTCGTTGACCTCCTCT/ACGGCACTGTAGGGTTCATC
	BBA_03436	hypothetical protein	GCGAAGAAGTCCTTTGCAGT/TCTTCTTGCACACACCAACC
	BBA_08602	LysM domain-containing protein	GTCATTTCTGCCGTTTTCGT/CCAAAGGTAAAGACGCCGTA
	BBA_07148	hypothetical protein	CCGCTACCTAAACGACCAGA/GTGGCGAGATCCTCGTAAAG
**CDA transcriptome**			
actB	BBA_04860	β-actin	GGCAACATTGTCATGTCTGG/TTTGCTGGAAGGTGGATAGG
	BBA_03106	xaa-pro dipeptidase	CCCTACTGGGATCCGTACAC/TTCATTAACCGGCCAACAGT
	BBA_09514	xanthine dehydrogenase	GCCCTGAAGCAGACTACTGG/CCTCACCCTCTGCAAAGAAG
	BBA_08144	hypothetical protein	GCCGAAGTCAGCATCTTTTC/TACACTCGGTGACGGTACGA
	BBA_03400	Cytochrome P450 CYP503B1	CACCCCGAGTACATTGAACC/GATTTCATGAACCGCCATCT
	BBA_06442	hypothetical protein	GGTGCCCTCTCTGTCTGTTC/GCTGCTGCTTGCAAATGATA
	BBA_05396	hypothetical protein	GGACTTTGCTACAGCCTTGG/TTCGTGTTCAAGGGGACTTC
	BBA_03331	lipase/serine esterase	ATCTATGCGTCCTCGTCCAC/CGAGTAGCCAATGATGCTGA
	BBA_06441	hypothetical protein	CGTCAATGACACCAACGATT/CACATTGACAAACCCAGCAG
	BBA_02211	ubiquitin-activating enzyme E1	CCTCTCCTCCCAATTCTTCC/ACGTAAATGCCCTTGGAGTG

* Gene accession codes in the *B. bassiana* genome under the NCBI accession NL_ADAH00000000.

Table S3. Transcript levels of Fluffy genes *flb*A–*flb*E in SDAY and CDA transcriptomes.

Gene	Tag loci*	Log_2FC(Rad6/ WT) SDAY	Padjust	Log_2FC(Rad6/ WT) CDA	Padjust
flbA	BBA_02968	**1.102**	< 0.001	**-1.367**	< 0.001
flbB	BBA_06988	**2.471**	< 0.001	0.461	< 0.001
flbC	BBA_01203	**-1.549**	< 0.001	**-1.883**	< 0.001
flbD	BBA_07259	0.736	< 0.001	**1.327**	0.003
flbE	BBA_01716	**1.253**	< 0.001	0.712	< 0.001
fluG	BBA_04942	**2.134**	< 0.001	-0.466	0.039
